# Smart Bandage Based on Molecularly Imprinted Polymers (MIPs) for Diclofenac Controlled Release

**DOI:** 10.3390/ph11040092

**Published:** 2018-09-22

**Authors:** Ortensia Ilaria Parisi, Mariarosa Ruffo, Luca Scrivano, Rocco Malivindi, Antonio Vassallo, Francesco Puoci

**Affiliations:** 1 Department of Pharmacy, Health and Nutritional Sciences, University of Calabria, 87036 Rende (CS), Italy; ortensiailaria.parisi@unical.it (O.I.P.); mariarosa.ruffo@unical.it (M.R.); luca.scrivano@unical.it (L.S.); rocco.malivindi@unical.it (R.M.); 2 Macrofarm s.r.l., C/O Department of Pharmacy, Health and Nutrition Sciences, University of Calabria, 87036 Rende (CS), Italy; 3 Department of Science, University of Basilicata, 85100 Potenza, Italy; antonio.vassallo@unibas.it

**Keywords:** Molecularly Imprinted Polymers (MIPs), Diclofenac, controlled release, smart bandage, in vitro diffusion studies, adsorption and release kinetics, skin irritation, precipitation polymerization

## Abstract

The aim of the present study was the development of a “smart bandage” for the topical administration of diclofenac, in the treatment of localized painful and inflammatory conditions, incorporating Molecularly Imprinted Polymers (MIPs) for the controlled release of this anti-inflammatory drug. For this purpose, MIP spherical particles were synthesized by precipitation polymerization, loaded with the therapeutic agent and incorporated into the bandage surface. Batch adsorption binding studies were performed to investigate the adsorption isotherms and kinetics and the selective recognition abilities of the synthesized MIP. In vitro diffusion studies were also carried out using Franz cells and the obtained results were reported as percentage of the diffused dose, cumulative amount of diffused drug, steady-state drug flux and permeability coefficient. Moreover, the biocompatibility of the developed device was evaluated using the EPISKIN™ model. The Scatchard analysis indicated that the prepared MIP is characterized by the presence of specific binding sites for diclofenac, which are not present in the corresponding non-imprinted polymer, and the obtained results confirmed both the ability of the prepared bandage to prolong the drug release and the absence of skin irritation reactions. Therefore, these results support the potential application of the developed “smart bandage” as topical device for diclofenac sustained release.

## 1. Introduction

Diclofenac (DC) is a phenylacetic acid derivative belonging to non-steroidal anti-inflammatory drugs (NSAIDs) and characterized by anti-inflammatory, analgesic and antipyretic activities [1,2,3]. This therapeutic agent is a non-selective inhibitor of the cyclooxygenase activity of prostaglandin H synthase [4] and it finds application in the treatment of inflammation and pain states related to several clinical conditions including arthritis, osteoarthritis, rheumatoid arthritis and acute gout, but also muscle injuries. 

The topical administration of diclofenac for the treatment of localized painful and inflammatory conditions has several advantages including avoiding first-pass hepatic metabolism and enzymatic degradation by the gastrointestinal tract and gastrointestinal irritation, but also the reduction of side effects and toxicity to other organs. Moreover, DC topical application using a bandage represents a less invasive option for the self-administration of this therapeutic agent. 

In this context, the development of a “smart bandage” for DC topical administration able to release the drug in a controlled way can be a further advance allowing the reduction in dosing frequency and, thus, dose related adverse effects. All these factors contribute to a better patient compliance. Therefore, the present research study exploited Molecular Imprinting Technology for the preparation of Diclofenac Imprinted Polymers to be used as drug delivery system incorporated into the bandage.

Molecularly Imprinted Polymers (MIPs) are synthetic polymers characterized by high selective recognition properties for a chosen target compound also in the presence of structurally similar molecules [5,6,7]. These systems are synthesized by polymerization of suitable functional monomers around the template molecule in the presence of a crosslinker in order to obtain a polymeric matrix characterized by specific binding cavities, which are complementary to the template in terms of size, shape and functionalities. The presence of these selective binding sites is due to the formation of a pre-polymerization complex between the template and the chosen functional monomers able to interact with the analyte via covalent or non-covalent way. Once the pre-polymerization complex is formed, the reaction occurs in the presence of the crosslinking agent leading to a polymeric material, which is subjected to an extraction process to remove the template molecule. 

This kind of polymeric materials finds application in a wide range of fields as stationary phase in chromatography, adsorbents in solid-phase extraction (SPE) procedures, synthetic receptors, antibodies and enzymes, and drug delivery systems (DDS). MIPs, indeed, can be used as drug carriers due to their ability to control the release of the therapeutic agent used as template during the polymerization process. This capability allows overcoming the drawbacks associated to a narrow therapeutic index reducing the side effects and improving the patient compliance. 

Based on these considerations, Diclofenac Imprinted Polymers for the sustained release of this anti-inflammatory agent were synthesized and incorporated into a bandage to prepare a novel smart dressing for the topical administration of DC by covering the textile with the drug loaded polymeric particles.

## 2. Results and Discussion

### 2.1. Adsorption Properties and Adsorption Kinetics

To investigate the imprinting efficacy of the prepared polymeric materials, binding experiments were performed incubating amounts of imprinted and non-imprinted particles in diclofenac standard solutions prepared in PBS at pH 7.4. 

The binding capacity of the synthesized MIP and NIP was evaluated considering the amount of DC bound to the polymers at equilibrium (*Q_e_*, mol/g), calculated according to Equation (1) [8]:

(1) $$\;Q_{e}=\frac{\left(C_{i}-C_{e}\right)\times \;V}{m}\;$$

where *C_i_* and *C_e_* (mol/L) are the initial and equilibrium concentrations of DC in solution, respectively, *V* (L) is the volume of the solution and *m* (g) is the weight of the polymers.

**Figure 1:**
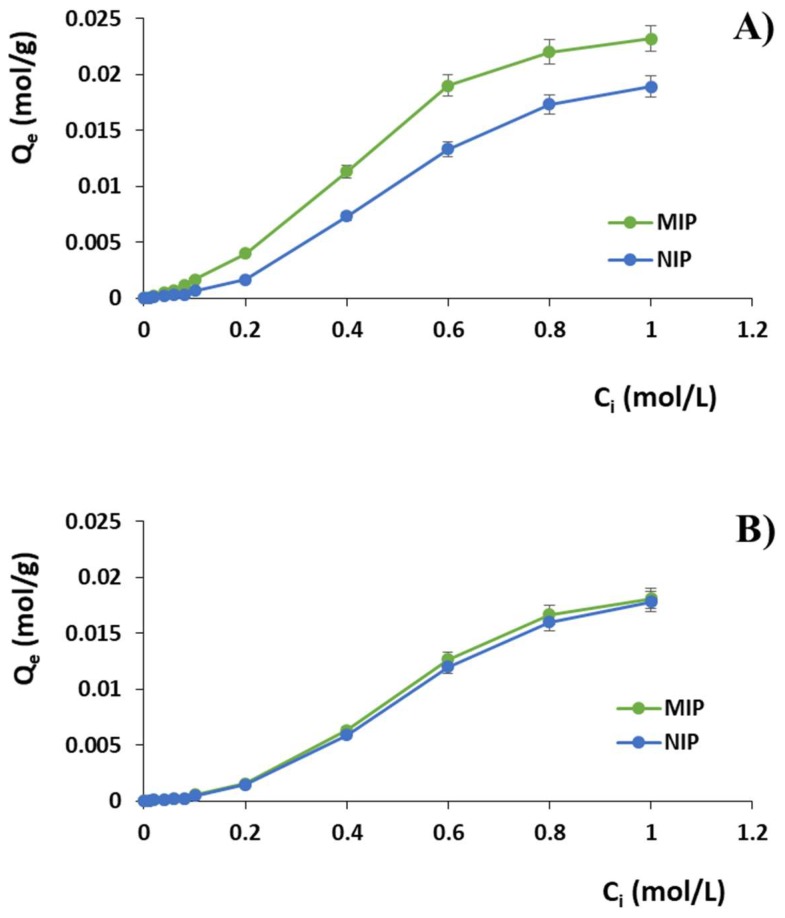
Adsorption isotherms of (**A**) DC and (**B**) PAA on MIP and NIP.

By plotting *Q_e_* versus *C_i_*, the adsorption isotherms of diclofenac on MIP and NIP were obtained, as shown in Figure 1A.

The same batch binding studies were performed using standard solutions of phenylacetic acid (PAA), which is a structural analogue of diclofenac, in order to evaluate also the selectivity properties of the imprinted polymer. The obtained isotherms curves are reported in Figure 1B, while Table 1 shows the observed percentages of bound diclofenac and phenylacetic acid by imprinted and non-imprinted polymers confirming both the imprinting effect and the selectivity of the prepared imprinted particles.

The imprinting factor (α) and the selectivity coefficient (ε) are also reported in Table 1. The first one is the ratio between the adsorption percentages of MIP and NIP for each analyte, such as the template (α DC) and its structural analogue (α PAA). The second coefficient represents the ratio between the adsorption percentages of DC and PAA observed for the imprinted polymer. 

**Figure 2:**
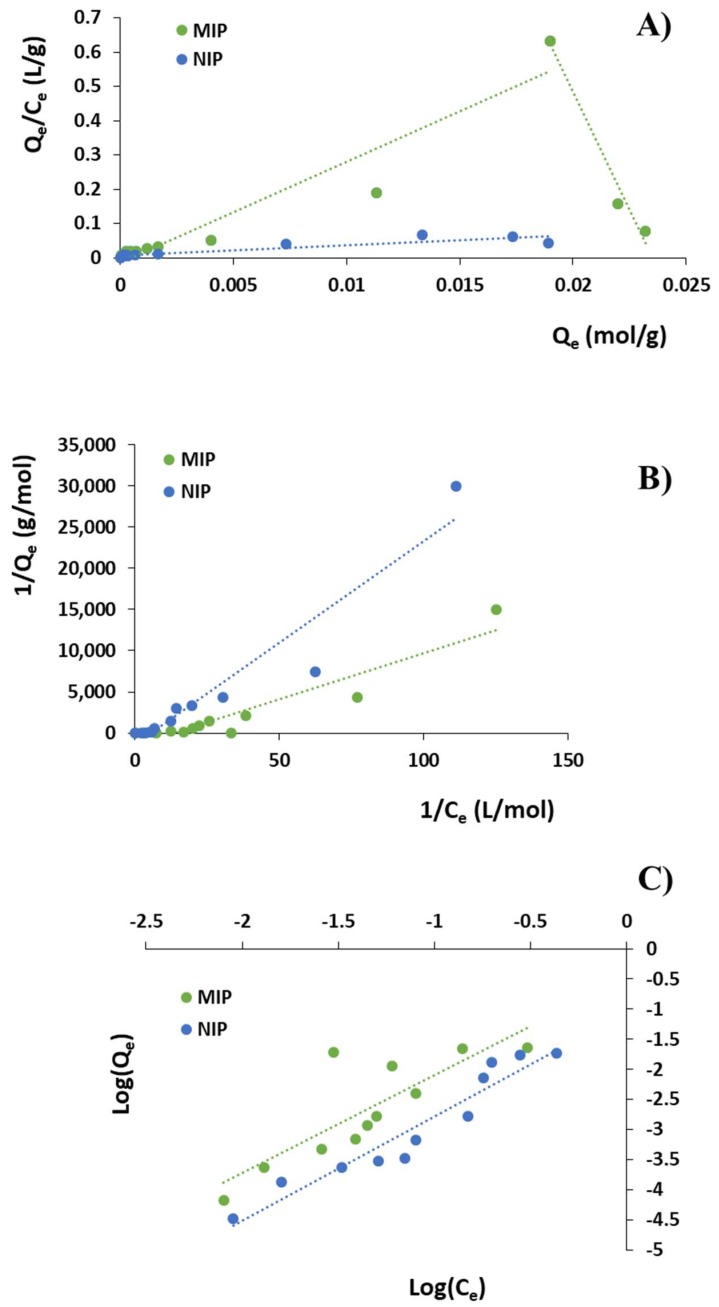
(**A**) Scatchard analysis; (**B**) Langmuir adsorption isotherms; and (**C**) Freundlich adsorption isotherms.

The *Scatchard model* is often used to characterize MIP adsorption properties and collect information about the affinity distribution of the binding sites discerning whether these sites are homogeneous or heterogeneous. Scatchard analysis was performed using Equation (2):

(2) $$\;\frac{Q_{e}}{C_{e}}=\left(B_{\max}-Q_{e}\right)K_{a}\;$$

where *Q_e_* is the amount of DC bound per gram of polymer at the equilibrium (mol/g), *C_e_* is the analyte equilibrium concentration (mol/L), *B*_max_ (mM/g) is the apparent maximum binding capacity and *K_a_* (M^−1^) is the association constant. By plotting *Q_e_*/*C_e_* versus *Q_e_*, *K_a_* and *B*_max_ of the polymer were determined from the slope and the intercept, respectively (Figure 2A).

As reported in Table 2, the high-affinity and low-affinity binding sites of MIP showed association constants (*K_a_*) equal to 137.64 and 29.48 M^−1^, respectively, while the apparent maximum binding capacity (*Q*_max_) of high-affinity and low-affinity binding sites were 23.50 and 0.49 mM/g.

To further study the adsorption properties of the synthesized polymeric particles, the obtained experimental data were fitted using the Langmuir and Freundlich models.

The *Langmuir model*, which represents the simplest model used in adsorption studies, involves a monolayer adsorption on a uniform surface containing a limited number of homogeneous binding sites able to accommodate one template molecule, meaning that no further adsorption can take place at that site [9]. This model assumes that the ability of the target molecule to bind at a given site is not affected by the occupation of nearby sites. Therefore, Langmuir isotherms are used to evaluate the maximum adsorption capacity, which is reached when surface achieves saturation, according to Equation (3):

(3) $$\;\frac{1}{Q_{e}}=\frac{1}{Q_{\max}C_{e}K_{L}}+\frac{1}{Q_{\max}}\;$$

where *Q_e_* is the amount of DC bound per gram of polymer at the equilibrium (mol/g), *C_e_* is the analyte equilibrium concentration (mol/L), *Q*_max_ is the maximum adsorption capacity corresponding to complete monolayer coverage on the surface (mol/g) and *K_L_* is the Langmuir constant related to the energy or net enthalpy of sorption (L/mol). By plotting 1/*Q_e_* versus 1/*C_e_* (Figure 2B), *K_L_* and *Q*_max_ of the polymer were determined (Table 3).

*Freundlich isotherm* allows better fitting the experimental data describing the surface heterogeneity of polymeric materials [10,11,12] and is expressed as Equation (4):

(4) $$\;Q_{e}=K_{F}C_{e}{}^{m}\;$$

where *K_F_* is the Freundlich constant related to the binding affinity ((mol/g)(mol/L)^m^) and *m* is the heterogeneity index (dimensionless), which ranges from 0 (for a heterogeneous system) to 1 (for a homogeneous system).

Equation (4) in its linear form is expressed as Equation (5):

(5) $$\;\log Q_{e}=\log K_{F}+m\log C_{e}\;$$

By plotting log*Q_e_* versus log*C_e_*, *K_F_* and *m* values can be determined (Figure 2C and Table 3). Freundlich isotherms are based on the adsorption on heterogeneous surface assuming that as the template concentration increases, the concentration of template on the adsorbent surface will increase. 

The *kinetics of adsorption* that describe DC uptake rate were investigated immersing 30 mg of polymeric particles in a 0.6 M solution of the drug and monitoring the therapeutic agent concentration at different time intervals. The amount of DC bound at time t (*Q_t_*, mol/g) was calculated by the difference between the initial drug concentration at *t* = 0 (*C_i_*, mol/L) and the residual concentration at the adsorption time *t* (*C_t_*, mol/L) according to Equation (6):

(6) $$\;Q_{t}=\left(C_{i}-C_{t}\right)\frac{V}{m}\;$$

where *V* is the volume of the incubation solution (L) and m is the amount of polymeric material (g).

To study the kinetic of DC adsorption on the imprinted polymer, two different models can be used such as the pseudo-first order and pseudo-second order models.

The first one is based on the assumption that one molecule adsorbs within the active site of the polymer [13] and it is given by Equation (7):

(7) $$\;\log\left(Q_{e}-Q_{t}\right)=\log\left(Q_{e}\right)-\frac{K_{1}}{2.303}t\;$$

where *Q_e_* is the amount of adsorbed drug at the equilibrium time, *K*_1_ is the first-order adsorption rate constant and *t* is the adsorption time.

The pseudo-second order model is based on the assumption that one adsorbate molecule interacts with two active sites [13] and is described by Equation (8):

(8) $$\;\frac{t}{Q_{t}}=\frac{1}{K_{2}Q_{e}{}^{2}}+\frac{1}{Q_{e}}t\;$$

where *K*_2_ is the pseudo-second order adsorption rate constant.

**Figure 3:**
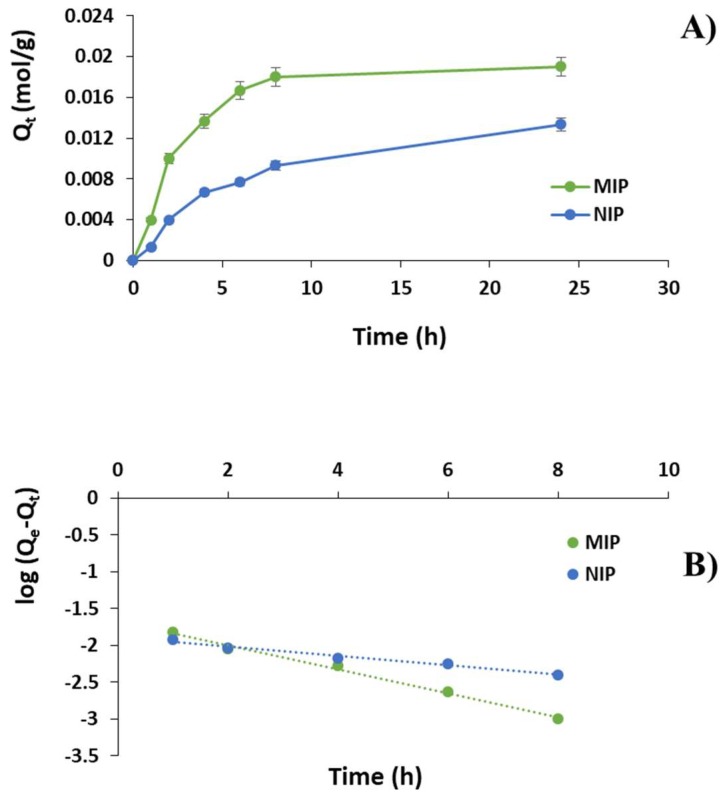

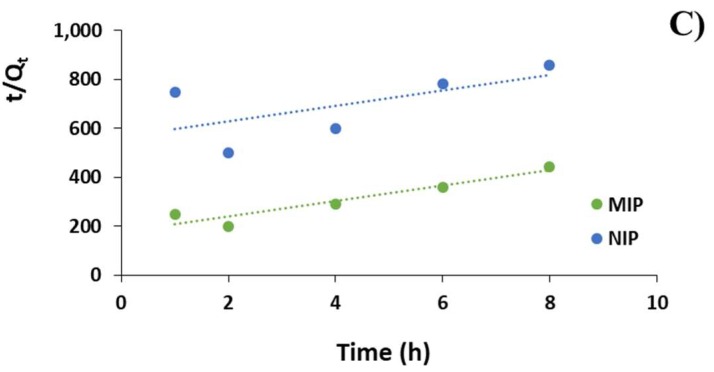
(**A**) DC adsorption kinetic curves for MIP and NIP; (**B**) DC adsorption kinetics according to pseudo-first order model; and (**C**) pseudo-second order model.

The adsorption kinetic curves for MIP and NIP are reported in Figure 3A by plotting *Q_t_* versus *t*.

The imprinted particles showed a fast template adsorption within the first 8 h followed by a plateau phase. After the first hour, indeed, 20% of DC was adsorbed by MIP reaching 68%, 83% and 90% at the time point of 4, 6 and 8 h, respectively. A plateau phase was observed between 8 and 24 h (95%). On the contrary, the amount of bound DC within the first hour was equal to 7% for the corresponding non-imprinted material getting 33%, 38% 47% and 67% at the time point of 4, 6, 8 and 24 h, respectively.

To investigate the adsorption mechanism, pseudo-first order and pseudo-second order kinetic models were adopted according to Equations (7) and (8), which are plotted in Figure 3B,C. The correlation coefficients and *Q_e_* values are reported in Table 4.

### 2.2. Drug Loading Content and Drug Loading Efficiency

The Drug Loading Content (DLC) and the Drug Loading Efficiency (DLE) were calculated according to Equations (9) and (10), respectively [14]:

(9) $$\mathrm{DLC}\;\left(\%\right)=\;\frac{\mathrm{weight}\;\mathrm{of}\;\mathrm{loaded}\;\mathrm{DC}}{\left(\mathrm{weight}\;\mathrm{of}\;\mathrm{loaded}\;\mathrm{DC}\;+\;\mathrm{weight}\;\mathrm{of}\;\mathrm{loaded}\;\mathrm{microspheres}\right)}\;\times 100\;$$

(10) $$\;\mathrm{DLE}\;\left(\%\right)=\;\frac{\mathrm{weight}\;\mathrm{of}\;\mathrm{loaded}\;\mathrm{DC}}{\mathrm{weight}\;\mathrm{of}\;\mathrm{DC}\;\mathrm{used}\;\mathrm{in}\;\mathrm{loading}\;\mathrm{procedure}}\times 100\;$$

DLC values were 7.1 ± 0.2% and 5.7 ± 0.5%, while DLE were 68.0 ± 0.4% and 54.0 ± 0.3% for MIP and NIP, respectively.

### 2.3. In Vitro Diffusion Studies

In vitro diffusion studies were carried out using Franz diffusion cells and a synthetic membrane-based model with diffusion characteristics correlated to human skin. The adopted experimental model was employed as a screening tool, without replacing human skin, to perform a pilot study of diffusion tests and collect preliminary data. 

The experiments were performed using two different bandages, which were prepared employing DC loaded MIP and NIP particles, respectively, and the obtained results were reported as percentage of the diffused dose, cumulative amount (*Q_t_*) of drug diffused through the membrane, steady-state drug flux (*J*) and permeability coefficient (*K_p_*) using receptor fluid data.

**Figure 4:**
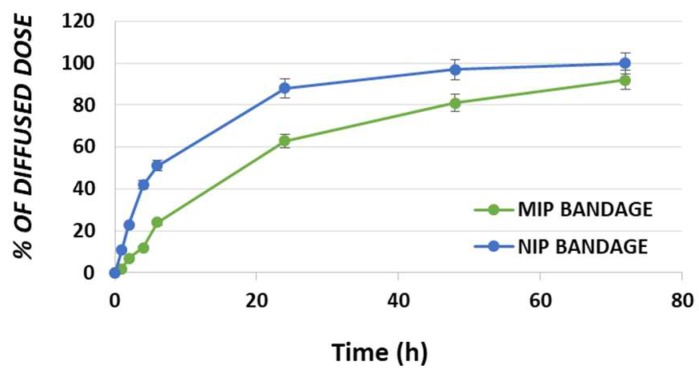
Percentage of DC diffused dose for MIP and NIP bandages.

**Figure 5:**
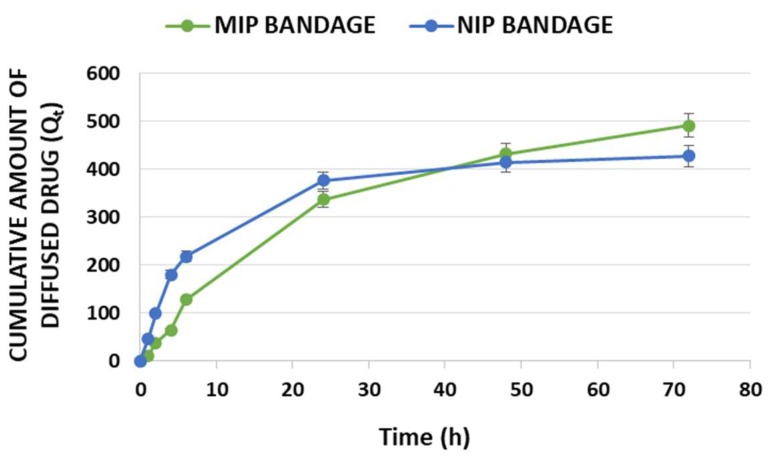
In vitro profiles of cumulative diffused amount *Q_t_* per unit area (μg/cm^2^) in time of diclofenac for MIP and NIP bandages.

The percentage of the diffused dose and the cumulative amount of diffused drug *Q_t_*, expressed as µg/cm^2^, for each tested item are reported in Figure 4 and Figure 5, respectively.

Other two significant parameters are represented by the steady-state flux (*J*) and the permeability coefficient (*K_p_*), which are reported in Table 5. for MIP and NIP based bandages.

Steady-state flux represents the amount of drug crossing the membrane at a constant rate, while the permeability coefficient is obtained from the relation between the flux and the initial concentration (*C_d_*) of diclofenac added to the donor compartment as reported in Equation (11):

(11) $$\;K_{p}=J/C_{d}\;$$

### 2.4. In Vitro Skin Irritation by EPISKIN™ Model

In the present study, the EPISKIN prediction model was employed according to OECD TG439 version 2015 to investigate the skin irritation potential of the developed smart bandage prepared using diclofenac imprinted particles.

**Figure 6:**
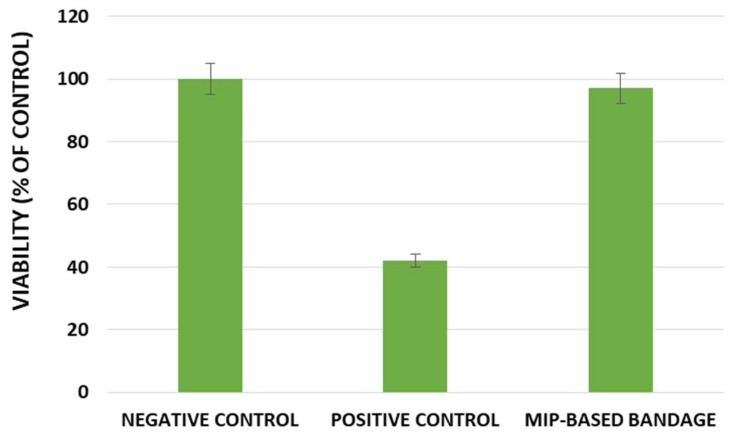
Effect of the developed smart bandage in EPISKIN™ RHE/L/13.

For this purpose, the in vitro reconstructed human epidermis was treated with 16 mg ± 2 mg (i.e., 32 mg/cm²) of bandage according to the “42 bis” procedure, which involves a 42 min exposure followed by a rinsing step and a 42 h post-incubation. Then, cell viability was evaluated by the MTT assay and the obtained results are reported in Figure 6.

## 3. Discussion

### 3.1. Diclofenac Imprinted Polymers and Their Adsorption Properties

As already reported in a previous work [15], the non-covalent approach for the synthesis of MIPs was chosen as the more appropriate to address our research purpose due to its advantages compared to the other ones. This synthetic methodology involves relatively weak non-covalent interactions, such as hydrogen bonds, dipole–dipole interactions, van der Waals forces and ionic interactions, which are formed between the selected template molecule and functional monomers both during the pre-polymerization and the rebinding steps. Therefore, an easy experimental procedure is required for both the imprinted polymers preparation and the subsequent removal of the template molecules, which leads to a polymeric matrix characterized by the presence of specific and highly selective recognition cavities. Moreover, an extensive range of monomers able to form pre-polymerization complexes with several template functionalities, including hydroxyl, carboxyl, amino and keto groups, is available. This makes possible to imprint a great variety of different analytes involving fast kinetics of recognition and binding.

Methacrylic acid and ethylene glycol dimethacrylate were selected as functional monomer and crosslinker, respectively. The first one is able to interact with diclofenac molecules by hydrogen bonds, while EGDMA stabilizes the polymer structure around the template. The employed DC/MAA/EGDMA molar ratio was the same identified in the previous study to obtain the best imprinting effect. Precipitation polymerization was adopted as simple method to obtain polymeric particles characterized by a spherical geometry. This technique consists of a single-step reaction carried out in a higher amount of porogenic solvent compared to bulk polymerization and allows exerting a good control of particle size. As reported in a previous work [15], the dimensional analyses of MIP highlighted a diameter of about 900 nm, while the binding cavities are complementary in size to the template and, thus, characterized by a smaller dimension.

Binding experiments were carried out to explore the imprinting efficacy and selectivity of the prepared polymeric materials. In particular, isotherms studies are useful to provide information about the nature of the template–polymer interaction and the obtained binding isotherms (Figure 1) showed that the adsorption abilities of the two compared polymers improved with the increasing diclofenac initial concentration *C_i_* until a saturation point. As it is possible to observe, the imprinted material is able to bind a higher amount of DC compared to the corresponding non-imprinted polymer. Moreover, the obtained α and ε values confirmed the selective binding properties for diclofenac of the imprinted particles compared to the corresponding non-imprinted material. The imprinting efficiency α, indeed, takes values greater than 1 for each adopted *C_i_*, which means that MIP is characterized by a higher capability to bind the drug compared to the corresponding NIP (Table 1). In addition, the obtained selectivity coefficient values indicate that MIP is from 1.28 to 4.38 times more selective for the drug than the corresponding control polymer in the adopted experimental conditions. 

The observed selective recognition ability of diclofenac imprinted polymers is due to the presence of specific binding sites into the polymeric matrix. These selective cavities are formed during the polymerization process carried out in the presence of the drug molecule and are complementary in terms of size, shape and electronic entourage with that of the template. On the contrary, non-specific interactions are responsible for the bound amount of PAA, which is equal for MIP and NIP particles.

The Scatchard model was used to characterize MIP adsorption properties and, as it is possible to observe in Figure 2A, the obtained MIP plot is not linear, but composed of two different straight lines; on the contrary, NIP plot consists of one line. The Scatchard analysis indicated that the recognition cavities of the imprinted polymer are not uniform in nature; therefore, MIP possesses heterogeneous binding sites consisting of two different types such as high-affinity and low-affinity ones. On the other hand, NIP showed a linear slope revealing homogeneous binding sites.

The obtained experimental data were also fitted using the Langmuir and Freundlich models (Figure 2B,C). As it is possible to observe from the Langmuir model, NIP presents a higher *Q*_max_ value than MIP (Table 3). This result is acceptable because this kind of model assumes that all the binding sites are homogeneous. However, this assumption is far from being true for all cases of Molecularly Imprinted Polymers that cannot fit so well to a homogeneous model. On the contrary, the Freundlich model proposes a non-ideal adsorption on heterogeneous surfaces, which is not restricted to the formation of a monolayer. 

Generally, MIPs present a higher degree of heterogeneity and, thus, a lower heterogeneity index (m) than the corresponding NIPs [16]. In the present study, Freundlich isotherm did not show a good linearity for DC adsorption on MIP (R^2^ equal to 0.69) and both imprinted and non-imprinted polymers showed m values above 1, which indicate a cooperative adsorption process that occurs when the binding of one molecule affects the binding of others [12]. 

Based on the values reported in Table 3, the obtained experimental data and, therefore, diclofenac adsorption on MIP better fit the Langmuir isotherm model with a higher R^2^ (0.88) indicating that the adsorption took place at specific homogeneous sites leading to a monolayer coverage of DC at the polymer surface.

The kinetics of DC adsorption were also investigated and, according to the correlation coefficients reported in Table 4, the experimental data fitted very well the pseudo-first order kinetic equations, which are characterized by correlation coefficients R^2^ of 0.99 and 0.98 and apparent adsorption rate constant *K*_1_ values of 0.38 and 0.15 for MIP and NIP, respectively. Moreover, the theoretical *Q_e_* values estimated from pseudo-first order kinetic model were equal to 0.02 and 0.01 mol/g for MIP and NIP respectively and, therefore, very close to the experimental ones. On the contrary, the pseudo-second order kinetic equations were not suitable to describe the adsorption kinetic due to correlation coefficients of only 0.90 and 0.40 for MIP and NIP, respectively. 

The obtained kinetic results indicated that the pseudo-first order adsorption mechanism was predominant in the adsorption process of diclofenac on the synthesized imprinted polymer and the higher MIP *K*_1_ value suggested that the adsorption rate of the imprinted material was apparently greater than that of the corresponding NIP. 

### 3.2. Drug Loading and “Smart Bandage” Preparation

The diclofenac loading process into the imprinted and non-imprinted materials is based on the physical diffusion of the drug molecules into the two synthesized polymeric matrices. This loading mechanism involves the formation of interactions between the drug molecules and the polymeric particles, which consist of hydrogen bonds and electrostatic and non-covalent interactions and depend on the functionalities exposed on template and polymers.

The Drug Loading Content (DLC) and the Drug Loading Efficiency (DLE) were calculated according to Equations (9) and (10) and the achieved values further confirmed the ability of the prepared imprinted particles to selectively interact with diclofenac due to the presence of specific binding sites.

In the present research study, diclofenac imprinted particles were synthesized and used for the preparation of a “smart bandage” for the topical treatment of inflammatory states involving joints, muscles, tendons or ligaments. For this purpose, a solution of polyvinyl alcohol (PVA) and polyvinyl pyrrolidone (PVP) K30, which are widely employed also in the textile industry, was used to allow the adhesion of DC loaded particles to the elastic bandage.

PVA was chosen for its water solubility and film forming properties [17,18], while PVP was selected due to its thickening and adhesive properties and the ability to form a flexible film [19,20]. The adopted PVA and PVP amounts were optimized to obtain an elastic bandage easily applicable around the affected area and, at the same time, to allow the effective adhesion of the drug loaded particles to the tissue avoiding the loss of polymeric material and, therefore, of the therapeutic potential of the final product. Lower amounts of PVA and PVP, indeed, exposed to the risk of loss of polymeric material; on the contrary, higher amounts made the tissue too rigid.

### 3.3. In Vitro diffusion Studies

The developed diclofenac delivery system has been designed for the treatment of localized pain, through topical application, which could be related to several inflammatory and clinical conditions including soft tissue musculoskeletal disorders, muscle injuries, arthritis, osteoarthritis, rheumatoid arthritis and acute gout. Therefore, the present study aims to increase the efficacy of the drug at the site of action. When the device is positioned in contact with the skin, the therapeutic agent migrates from the device, it is partitioned across the device/skin interface and, finally, migrates into the skin to reach the site of action.

The in vitro diffusion experiments were performed using two different bandages, which were prepared using DC loaded MIP and NIP particles respectively, and a synthetic membrane was employed instead of human skin. The correlation to human skin and the use of Strat-M^®^ was widely reported in literature suggesting that this kind of experimental model could be employed as screening tool to perform a pilot study of diffusion tests collecting preliminary data [21,22].

As it is possible to observe in Figure 4, the drug diffusion process is characterized by three different steps such as burst, time delay and stable diffusion. The bandage prepared using DC loaded NIP particles showed a percentage of the diffused dose equal to 51% within the first 6 h reaching the 88%, 97% and 100% in 24, 48 and 72 h, respectively. On the other hand, the bandage incorporating DC loaded MIP particles exhibited a value of 24% after the first 6 h and achieving 63%, 81% and 92% at the time points of 24, 48 and 72 h, respectively.

**Scheme 1:**
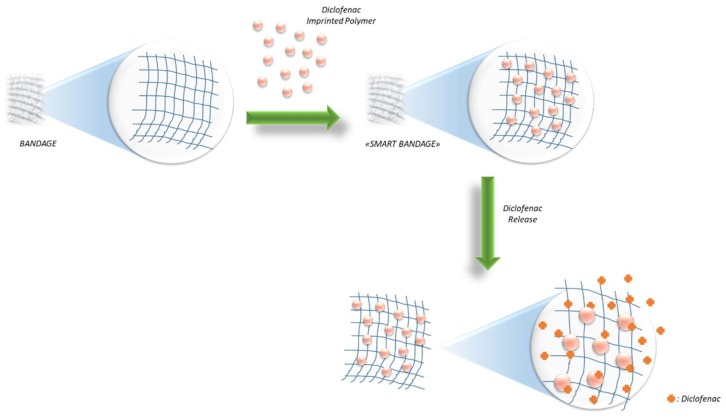
Schematic illustration of MIP-based bandage preparation and diclofenac release.

The obtained profiles confirmed the drug prolonged diffusion through the membrane and, therefore, the prolonged drug release from the smart bandage prepared using DC imprinted polymer due to the presence of specific binding sites able to interact strongly with the drug molecules (Scheme 1). 

In Figure 5, the cumulative amount (*Q_t_*) of diffused diclofenac is reported. The obtained in vitro profiles are due to the different polymeric matrices employed in the preparation of the two tested textiles. The imprinted polymer is able to load a higher amount of drug compared to the non-imprinted one in the same experimental conditions. This explains the observed *J* and *K_p_* values (Table 5) and why the cumulative amount of diffused DC at 72 h is higher for the MIP-based bandage even though the percentages of diffused dose at the same time were 92% and 100% for MIP and NIP textiles, respectively. 

### 3.4. In Vitro Skin Irritation by EPISKIN*™* Model

The EPISKIN prediction model was used to evaluate the skin irritation potential of the developed smart bandage prepared using the DC imprinted particles. The obtained results in the terms of cell viability, reported in Figure 6, confirmed the absence of irritation effects for the smart bandage.

## 4. Materials and Methods

### 4.1. Materials

Diclofenac sodium salt (DC), methacrylic acid (MAA), ethylene glycol dimethacrylate (EGDMA), 2,2′-azoisobutyronitrile (AIBN), phenylacetic acid (PAA), poly(vinyl alcohol) (PVA), polyvinylpyrrolidone (PVP, K30), disodium hydrogen phosphate, sodium dihydrogen phosphate, ammonium acetate, acetic acid, and 3-[4,5-di-methyl-thiazol-2-yl]-2,5-diphenyl tetrazolium bromide (MTT) were purchased from Sigma-Aldrich (Milan, Italy).

The functional monomer and the radical initiator were purified before use by a single-step passage through an alumina column and recrystallization in methanol, respectively.

All solvents were reagent or HPLC grade and purchased from VWR (Milan, Italy).

Strat-M^®^ membranes (25 mm discs, Cat. No. SKBM02560) were purchased by Merck Millipore.

*Flexa Elast* universal elastic bandage 5 cm × 4.5 m was purchased by Pic^®^.

The EPISKIN™ RHE/L/13 human skin equivalent kit was obtained from SkinEthic Laboratories (Lyon, France).

### 4.2. Instrumentation

The HPLC analyses were carried out using a Jasco PU-2080 liquid chromatograph (Tokyo, Japan) equipped with a Rheodyne 7725i injector (fitted with a 20-µL loop), a Jasco UV-2075 HPLC detector and a Jasco-Borwin integrator (Massachusetts, USA). The adopted HPLC conditions for diclofenac analysis have been previously reported in the literature [15].

In vitro diffusion studies were carried out using Franz diffusion cells (Disa, Milan, Italy; permeation area 0.4614 cm^2^).

### 4.3. Synthesis of Molecularly Imprinted Polymers (MIPs)

Diclofenac Molecularly Imprinted Polymers were synthesized via precipitation polymerization method using methacrylic acid, ethylene glycol dimethacrylate and 2,2′-azoisobutyronitrile as functional monomer, crosslinking agent and radical initiator, respectively. The adopted reaction conditions have been reported in a previous work [15].

To remove DC template molecules, the extraction procedure was carried out using a Soxhlet apparatus and an acetic acid solution in methanol (10% *v*/*v*) for the first 24 h followed by methanol for other 24 h as extraction solvents. Finally, the polymeric particles were dried overnight at 40 °C.

The corresponding Non-Imprinted Polymers (NIPs) were also prepared following the same experimental procedure but in the absence of the template molecule.

### 4.4. Batch Adsorption Binding Studies

The binding studies were designed to verify both the imprinting efficiency and the selectivity of the synthesized polymeric particles. 

The experiments were carried out in phosphate buffer solution at pH 7.4 (10^−3^ M) and using 30 mg of dried polymeric particles, which were mixed with 1 mL of DC standard solution (0.01–1.0 M). After 24 h, the samples were centrifuged at 10,000 rpm for 20 min and the drug concentration was quantified by HPLC analysis.

Similar binding experiments were carried out with phenylacetic acid (PAA) to evaluate the selectivity of the prepared imprinted polymer.

The binding protocol was repeated three times.

### 4.5. Kinetic Adsorption Binding Studies

The adsorption kinetics were investigated by batch adsorption experiments in which 30 mg of polymeric particles were mixed with 1 mL of a DC standard solution (0.6 M) prepared in phosphate buffer at pH 7.4 (10^−3^ M). DC concentration was monitored by HPLC analysis at different incubation times (1–24 h), after which each sample was centrifuged at 10,000 rpm for 20 min.

The experiments were repeated in triplicate.

### 4.6. Drug Loading Procedure

In a 10 mL round-bottom flask, 180 mg of the synthesized polymeric imprinted and non-imprinted particles were immersed in 2 mL of a 31.4 mM diclofenac solution previously prepared using an acetonitrile/methanol mixture (1:1 *v*/*v*). The samples were soaked under continuous stirring in dark conditions at room temperature; after three days, the polymeric particles were transferred into sintered glass filters in order to remove the solvent by percolation and, finally, dried under vacuum at 40 °C overnight.

The leachate was analyzed for the quantification of unloaded DC by HPLC and Drug Loading Content (DLC) and Drug Loading Efficiency (DLE) were calculated according to Equations (9) and (10), respectively.

### 4.7. “Smart Bandage” Preparation

For the preparation of the “Smart Bandage”, 30 mg of the drug loaded particles were dispersed in 0.5 mL of a PVA aqueous solution (4% *w*/*v*) containing 12.5 mg of PVP. The obtained dispersion was used to coat in a uniform way the surface of a 2 cm × 2 cm portion of the elastic bandage, which was then dried in an oven at 55 °C for one hour.

Two different smart bandages were prepared using loaded MIP and NIP particles, respectively.

### 4.8. In Vitro Diffusion Studies

The efficacy of the developed “Smart Bandage” was investigated by performing in vitro diffusion studies. All experiments were conducted at 37 ± 0.5 °C and using Strat-M^®^ membranes, which were placed between the donor and the receptor compartments of the Franz diffusion cells [23].

The smart bandage was positioned on the Strat-M^®^ membrane with the particles layer facing towards the acceptor compartment; then, the two chambers were fixed together. The donor and receptor compartments were filled with 0.5 and 5.5 mL of phosphate buffer at pH 7.4 (10^−3^ M), respectively. The content of the receptor chamber was removed at several time intervals (1, 2, 4, 6, 24, 48 and 72 h) for HPLC analysis and replaced with fresh phosphate buffer.

The experiments were performed in triplicate using two different bandages, which were prepared employing DC loaded MIP and NIP particles, respectively. 

The obtained results were reported as percentage of the diffused dose, cumulative amount (*Q_t_*) of drug diffused through the membrane, steady-state drug flux (*J*) and permeability coefficient (*K_p_*) using receptor fluid data.

### 4.9. In Vitro Skin Irritation by EPISKIN™ Model

In the aim to investigate the skin irritation potential of the developed bandage incorporating Diclofenac Imprinted Polymers, the EPISKIN™ RHE/L/13 human skin was treated with 16 mg ± 2 mg (i.e., 32 mg/cm²) of bandage, phosphate buffer as negative control and SDS (5% *w*/*v*) as positive control following the OECD TG439 version 2015 according to the “42 bis” procedure [23]. 

The experiments were carried out in triplicate.

## 5. Conclusions

The present study was focused on the development of a bandage incorporating Molecularly Imprinted Polymers for the topical administration of an anti-inflammatory drug such as diclofenac.

Batch binding studies were carried out to explore the adsorption isotherms and kinetics and confirmed the selective recognition abilities of the prepared DC imprinted particles. The Scatchard analysis indicated that the synthesized MIP is characterized by the presence of specific binding sites for the therapeutic agent, which are not present in the corresponding non-imprinted polymer. The kinetic data were also analyzed showing that diclofenac adsorption on MIP better fit the Langmuir isotherm model.

The performed in vitro diffusion studies demonstrated the ability of the prepared dressing to release the therapeutic agent in a controlled manner due to the presence of diclofenac imprinted particles. The observed drug diffusion, indeed, appears to be prolonged over time reaching an amount of released DC equal to 92% within 72 h. The achieved DLC and DLE values confirmed the good loading ability of MIP particles due to the presence of specific binding cavities, which make this polymeric material a suitable carrier. 

Moreover, the employed EPISKIN model confirmed the absence of skin irritation potential and, therefore, the bioavailability of the developed MIP-based bandage. 

Therefore, these results support the potential application of the developed “smart bandage” as topical device for diclofenac sustained release.

## Figures and Tables

**Table 1:** Percentages of bound diclofenac (DC) and phenylacetic acid (PAA) by imprinted (MIP) and non-imprinted (NIP) polymers and α and ε values for different *C_i_*. Data are shown as means ± S.D.

*C_i_* (mol/L)	BOUND DC (%)		BOUND PAA (%)		α DC	α PAA	ε
	MIP	NIP	MIP	NIP			
0.01	20.0 ± 0.8	10.0 ± 0.5	7.0 ± 0.4	5.6 ± 0.9	2.00	1.25	2.86
0.02	35.0 ± 0.6	20.0 ± 0.6	16.5 ± 0.7	12.9 ± 0.7	1.75	1.28	2.12
0.04	35.0 ± 0.3	17.5 ± 0.9	12.5 ± 0.7	9.5 ± 0.4	2.00	1.32	2.80
0.06	35.0 ± 0.7	15.0 ± 0.4	11.7 ± 0.8	9.3 ± 0.6	2.33	1.25	3.00
0.08	43.8 ± 0.4	12.5 ± 0.5	10.0 ± 0.4	6.9 ± 0.7	3.50	1.45	4.38
0.1	50.0 ± 0.6	20.0 ± 0.3	17.0 ± 0.7	14.0 ± 0.6	2.50	1.21	2.94
0.2	60.0 ± 0.6	25.0 ± 0.7	23.5 ± 0.5	22.0 ± 0.5	2.40	1.07	2.55
0.4	85.0 ± 0.5	55.0 ± 0.5	47.5 ± 0.6	44.3 ± 0.8	1.55	1.07	1.79
0.6	95.0 ± 0.8	66.7 ± 0.6	63.3 ± 0.4	60.0 ± 0.5	1.43	1.06	1.50
0.8	82.5 ± 0.4	65.0 ± 0.5	62.5 ± 0.8	60.0 ± 0.7	1.27	1.04	1.32
1.0	69.6 ± 0.7	56.7 ± 0.5	54.3 ± 0.6	53.5 ± 0.4	1.23	1.01	1.28

**Table 2:** *K_a_*, *B*_max_ and R^2^ values obtained by Scatchard analysis.

POLYMER	High Affinity Sites			Low Affinity Sites		
	*K_a_* (M^−1^)	*B*_max_ (mM/g)	R^2^	*K_a_* (M^−1^)	*B*_max_ (mM/g)	R^2^
***MIP***	137.64	23.50	0.98	29.48	0.49	0.91
***NIP***	-	-	-	3.05	2.13	0.84

**Table 3:** *K_L_*, *Q*_max_ and *R*^2^ and *K_F_*, m and R^2^ values obtained by Langmuir and Freundlich models, respectively.

POLYMER	LANGMUIR MODEL			FREUNDLICH MODEL		
	*K_L_* (L/mol)	*Q*_max_ (mmol/g)	R^2^	*K_F_*	M	R^2^
***MIP***	13.29	0.67	0.88	0.33	1.63	0.69
***NIP***	5.72	0.71	0.92	0.09	1.74	0.92

**Table 4:** Kinetic fitting data for MIP and NIP.

POLYMER	PSEUDO-FIRST ORDER			PSEUDO-SECOND ORDER		
	*K*_1_	*Q_e_*	R^2^	*K*_2_	*Q_e_*	R^2^
***MIP***	0.38	0.02	0.99	6.30	0.03	0.90
***NIP***	0.15	0.01	0.98	1.97	0.03	0.40

**Table 5:** Permeation parameters of diclofenac for MIP and NIP bandages: steady-state flux (*J*) and permeability coefficient (*K_p_*).

MIP BANDAGE		NIP BANDAGE	
*J* (μg/cm^2^ h)	*K_p_* × 10^−3^ (cm/h)	*J* (μg/cm^2^ h)	*K_p_* × 10^−3^ (cm/h)
8.0 ± 0.3	16.3 ± 0.6	7.6 ± 0.5	19.3 ± 0.4
